# First person – Roberta Besio

**DOI:** 10.1242/dmm.040865

**Published:** 2019-06-20

## Abstract

First Person is a series of interviews with the first authors of a selection of papers published in Disease Models & Mechanisms (DMM), helping early-career researchers promote themselves alongside their papers. Roberta Besio is first author on ‘Cellular stress due to impairment of collagen prolyl hydroxylation complex is rescued by the chaperone 4-phenylbutyrate’, published in DMM. Roberta is a postdoc in the lab of Antonella Forlino at University of Pavia, Italy, investigating collagen and genetic diseases of the connective tissue.


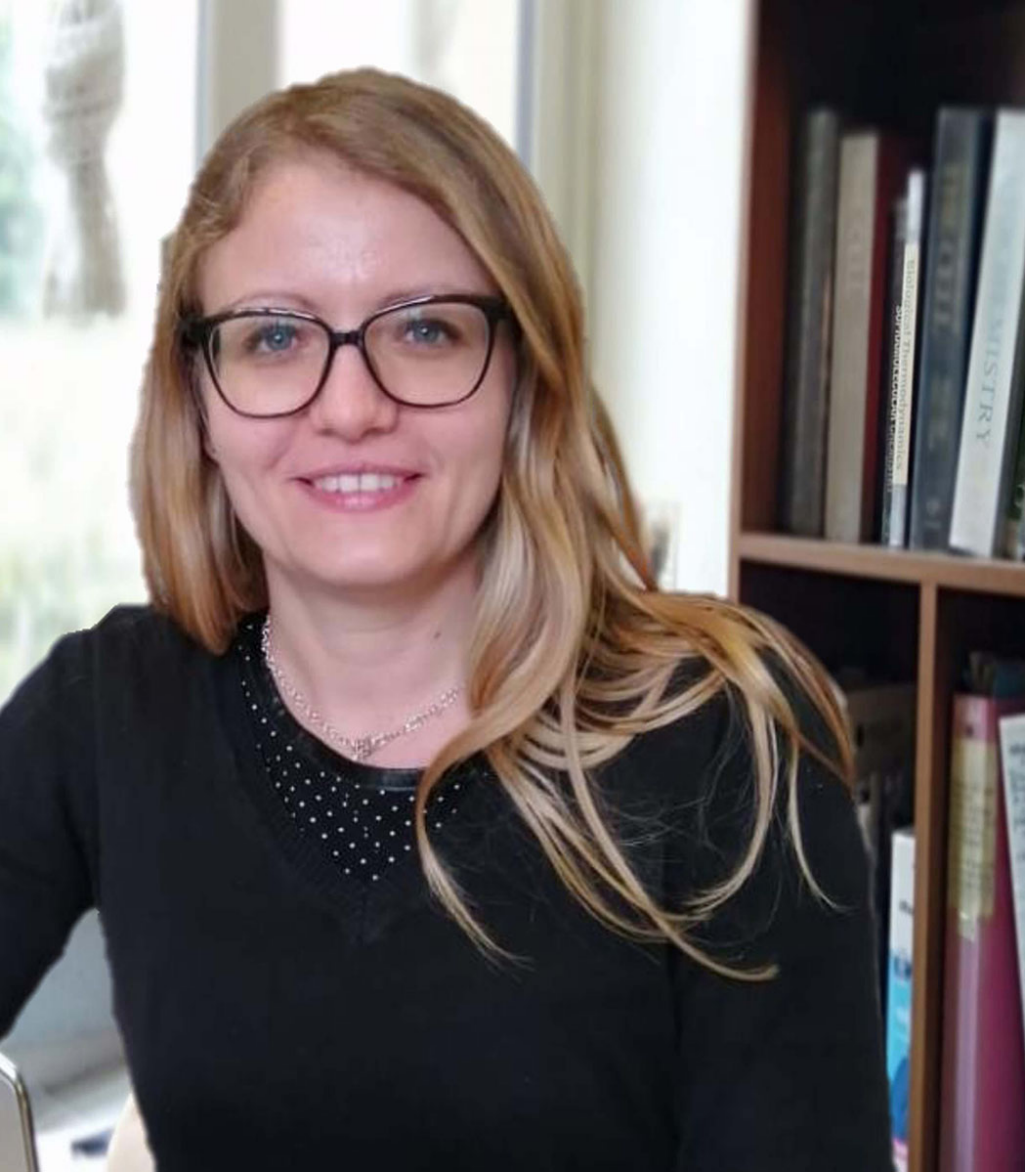


**Roberta Besio**

**How would you explain the main findings of your paper to non-scientific family and friends?**

Bone is mainly made up of collagen and contains cells that form and degrade bone to maintain its strength. Our study aimed to understand the role of cell activity on bone fragility, which is typically present in aged people as well as in people affected by heritable skeletal diseases. We focused our investigation on osteogenesis imperfecta (OI; brittle bone disease), a rare genetic incurable skeletal disorder. In kids with OI, cells produce an altered collagen molecule that is abnormally retained, causing cellular engulfment that leads to stress and sometimes also to cellular death. We treated the pathologic cells with a drug, 4-phenyl butyric acid (4-PBA), that was able to successfully alleviate the stress and to reduce cell death. We demonstrated that the activity of the drug is given by its ability to assist proteins to reach their correct structure, helping the clearance of the cell. Our findings demonstrate a new potential target for OI therapy and identified 4-PBA as a possible OI drug candidate. Furthermore, we demonstrated that the same cellular response occurs in different OI forms and, thus, that the proposed treatment can be effective on a higher number of individuals affected by this rare disease.

“Our findings demonstrate a new potential target for OI therapy and identified 4-PBA as a possible OI drug candidate.”

**What are the potential implications of these results for your field of research?**

The molecular basis of OI and of several bone diseases was completely attributed to the presence of an abnormal extracellular matrix and impairment of its structural integrity. It is now clear that mutated proteins are intracellularly retained and accumulate in the endoplasmic reticulum (ER), causing cellular stress that, in turn, contributes to the skeletal outcomes. Indeed, collagen type I misfolding and intracellular accumulation, as well as dilation of the ER, have been observed in OI, both in patients' cells and in various OI murine models. Here, we demonstrated that the cellular outcome in primary fibroblasts from patients with recessive forms of the disease is significantly affected by stress and malfunction caused by the retention of altered collagen and, thus, that the cellular response to collagen retention, together with the altered matrix, is a key component in OI pathogenesis. Furthermore, we demonstrated that the stress response can be tuned by a chaperone-based therapy, similarly to what we recently reported for dominant OI caused by mutations in collagen coding genes. Thus, our data opens the appealing possibility to have the same target for the OI forms characterized by the presence of overmodified collagen, allowing a common treatment. Together with the canonical anabolic and anti-catabolic drugs, 4-PBA should be considered to face the disease from a different, complementary, prospective.

**What are the main advantages and drawbacks of the model system you have used as it relates to the disease you are investigating?**

As model of the disease we used primary fibroblasts from recessive OI patients, since skin biopsy has limited invasiveness, allowing us to collect samples from several cases. Furthermore, a large body of literature is available on OI biochemical characterization based on this cell type, and fibroblasts share with osteoblasts the production of a high amount of collagen type I and several biochemical pathways. Furthermore, a skin phenotype is often described in OI patients. Nevertheless, OI is mainly a bone disorder and the bone-forming cells are known to produce an even higher amount of collagen type I, with a higher glycosylation level compared to fibroblasts. Further investigation in murine and, even better, in human osteoblasts will complement the data.

**Figure:**
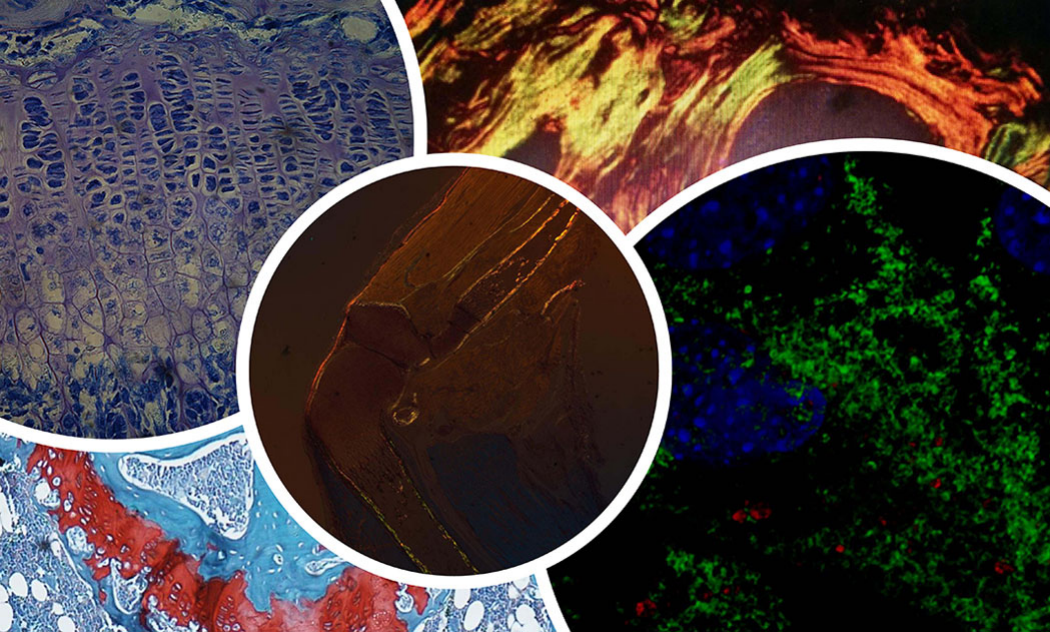
**Toluidine Blue and Masson's Trichrome staining of growth plate; Sirius Red staining of collagen in bone lamellae and in all limbs; and collagen immunofluorescence in osteoblasts.**

The idea to perform our study *in vitro* originated from the rationale of dissecting the involvement of pathways that are difficult to study *in vivo*. However, we are aware that there are limitations in *in vitro* systems: i.e. no information about the feedback of the mutant matrix on the cell. Indeed, transgenic mouse models for several proteins involved in the pathways we are interested in (Chop, Bip, etc.) are available as alternatives to the cellular system, and will hopefully allow us to further dissect the molecular bases of the disease.

**What has surprised you the most while conducting your research?**

I was amazed by the extreme cellular insult by mutant collagen retention, which caused not only impaired cell activity but cell death. I was very surprised when I realized that the chemical chaperone 4-PBA is able to rescue cellular homeostasis and to reduce cell death. Although in OI the extracellular matrix is compromised, acting on cellular pathways is so beneficial for the cell to positively act on its fate.

**Describe what you think is the most significant challenge impacting your research at this time and how will this be addressed over the next 10 years?**

No definitive therapy is available for the bone disease OI. Currently, physiotherapy, rehabilitation, orthopedic surgery and oral administration of the anti-resorption drug bisphosphonates are the treatments of choice to improve OI patients' lifestyles. Recently, efforts have been taken to develop both anti-catabolic and anabolic drugs to improve bone quality. With our research we identified in cellular stress a new target, whose modulation can significantly affect cellular homeostasis and thus cellular health. I think that the most significant challenge in the next 10 years will be to try to face the disease from two sides: the first is to develop a combined therapy of drugs able to stimulate osteoblasts activity, to inhibit osteoclasts activity and to restore cellular homeostasis, possibly targeting specifically the bone tissue; the second is to correct the causative molecular defect, the only action capable of really curing the disease. The advance in gene-editing strategies, thanks to the discovery of the CRISPR/Cas technique, really opens up new possibilities if flanked by innovative delivery systems.

“The advance in gene-editing strategies, thanks to the discovery of the CRISPR/Cas technique, really opens up new possibilities if flanked by innovative delivery systems.”

**What changes do you think could improve the professional lives of early-career scientists?**

The early-career scientist lives in a transition period with no certainty and high pressure to reach project goals and to get a stable position. An early-career scientist looks for relevant questions to answer and needs the possibility to discuss data and the progress of the project with mentors and experts in the field to get advice, a dynamic environment to work in and funding to pursue her/his goals. Increasing the funding opportunities for early-career scientists would indeed improve our professional lives, since this will help us to become independent and to gain credibility in the field. Getting your own funding encourages and empowers the scientist and can definitely help to successfully make the transition phase. Being the principal investigator of a research project gives the opportunity to approach the study from a different perspective, to face the challenges of choosing the most appropriate experiments to achieve the goals and to personally tackle the resolution of the problems encountered in the various phases of the experimental activity. Also, the opportunity to become responsible for the activities of Masters and PhD students will help develop supervisor skills that I consider fundamental for the next phase. Being exposed to an international environment and having the possibility to attend conferences, thanks to reduced registration fees or travel grants, will foster relationships outside the group and will help to establish collaborations, which are vital in our work.

“The early-career scientist lives a transition period with no certainty and high pressure to reach the project goals and to get a stable position.”

**What's next for you?**

Currently I am a postdoctoral fellow and I managed to obtain my first grant as principal investigator through the Cariplo Foundation. I recently applied for a stable position in my current university and I am waiting for the evaluation. Since the grant will expire soon, in the meantime I am trying to collect our last data for a new application. My long-term goal is to pursue a career in academia and to try to contribute to advances in the bone diseases field, and likely in the cure for OI.
